# Supplementary figures and images for: Effects of gene–lifestyle interactions on obesity based on a multi-locus risk score: A cross-sectional analysis
Source: PLoS One. 2023 Feb 8;18(2):e0279169. doi: 10.1371/journal.pone.0279169 (PMC9907830; doi:10.1371/journal.pone.0279169)

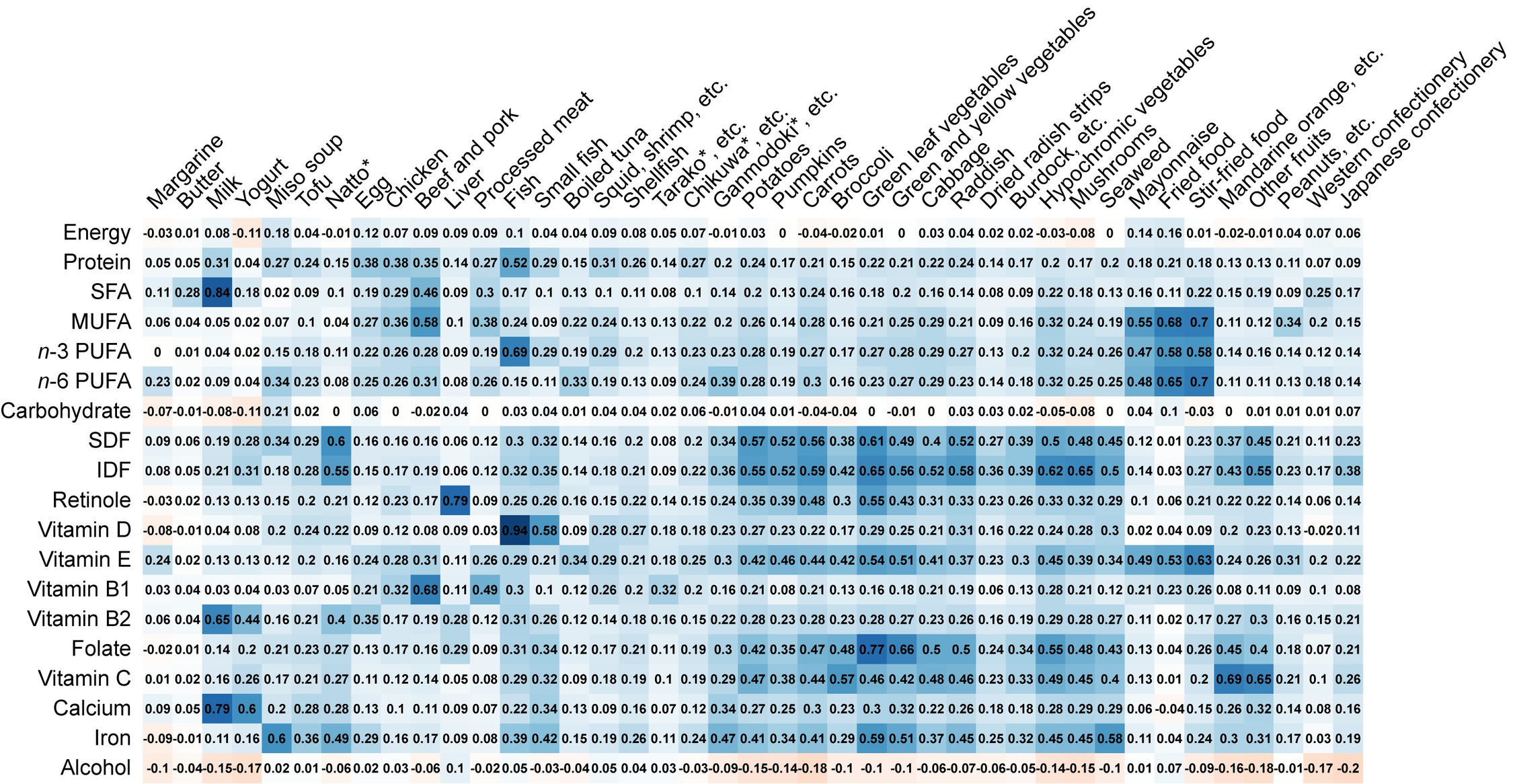

Supplement: S1 Fig — Each number in the matrix indicates correlation coefficients for each pair of nutrients (rows) and foods (columns). *Natto is fermented soybeans, tarako is a salted sack made from pollock or cod roe, chikuwa is a processed fish paste, and ganmodoki is deep-fried tofu fritters. SFA, saturated fatty acids; MUFA, monounsaturated fatty acids; n-3 PUFA, n-3 polyunsaturated fatty acids; n-6 PUFA, n-6 polyunsaturated fatty acids; SDF, soluble dietary fiber; IDF, insoluble dietary fiber. (TIF) [file pone.0279169.s001.tif]

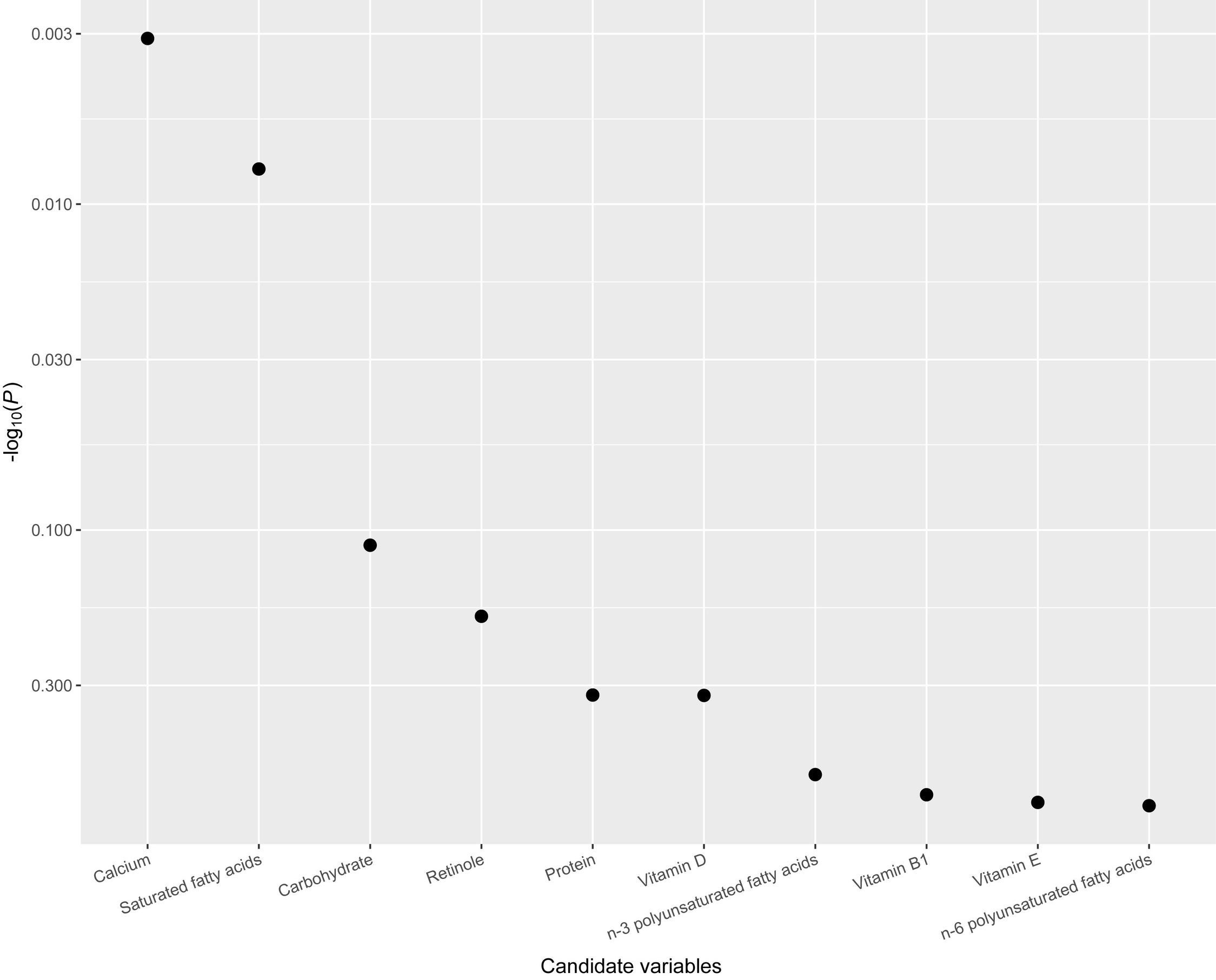

Supplement: S2 Fig — Each dot indicates the p-value for the interaction terms between GRS and each candidate variable in the linear-mixed model. The dependent variable of the model was BMI, with a recruited site-specific random intercept, and the fixed effect was age, sex, GRS, the interaction term between age and sex, and the interaction term between GRS and candidate variable. GRS, genetic risk score. (TIF) [file pone.0279169.s002.tif]
