# Supplementary material for: Effects of gene–lifestyle interactions on obesity based on a multi-locus risk score: A cross-sectional analysis
Source: PLoS One. 2023 Feb 8;18(2):e0279169. doi: 10.1371/journal.pone.0279169 (PMC9907830; doi:10.1371/journal.pone.0279169)
Supplement: S1 Table — (PDF) [file pone.0279169.s003.pdf]

S1 Table. BMI-associated loci in the Japanese population.

SNP	Position (bp)[a]	Chromosome	Reference allele	Alternative allele	Gene	Beta coefficients (SEM)[b]	P value	Inclusion in the GRS calculation	r^2	Effect allele frequency
rs2076463	27,971,092	1	A	G	FGR,IFI6	-0.023 (0.004)	1.68E-08	Included	0.97	0.28
rs860295	155,767,708	1	A	G	GON4L	0.029 (0.004)	8.66E-11	Included	0.95	0.25
rs633715	177,852,580	1	T	C	LOC101928778,SEC16B	0.049 (0.004)	1.05E-33	Included	0.999	0.23
rs491055	190,308,834	1	A	G	BRINP3	-0.022 (0.004)	2.82E-10	Included	0.995	0.35
rs939584	621,558	2	C	T	FAM150B,TMEM18	0.056 (0.006)	6.40E-23	Included	0.998	0.10
rs111612372	20,433,218	2	T	C	SDC1,PUM2	-0.033 (0.006)	2.33E-08	Excluded	-	-
rs713586	25,158,008	2	T	C	ADCY3,DNAJC27	0.025 (0.003)	4.92E-13	Included	0.997	0.48
rs6734118	37,559,355	2	C	A	PRKD3,QPCT	0.024 (0.003)	3.42E-12	Included	0.997	0.46
rs77489951	38,750,287	2	C	T	LOC101929596,HNRNPLL	0.044 (0.008)	9.39E-09	Included	0.91	0.06
rs10174398	51,195,601	2	T	C	NRXN1	-0.020 (0.003)	4.65E-09	Included	0.99	0.41
rs10208649	54,161,363	2	T	C	PSME4	-0.111 (0.016)	4.95E-12	Included	0.98	0.01
rs10197655	58,791,420	2	G	A	LINC01122	-0.020 (0.003)	3.63E-09	Included	0.999	0.41
rs12617004	142,615,136	2	G	C	LRP1B	0.020 (0.004)	5.83E-09	Included	0.99	0.39
rs2390669	169,091,942	2	A	C	STK39	0.024 (0.004)	5.63E-10	Included	1.000	0.23
rs6433857	181,517,996	2	C	T	CWC22,SCHLAP1	-0.021 (0.004)	4.57E-08	Included	0.93	0.32
rs2574704	11,655,381	3	T	C	VGLL4	0.019 (0.004)	4.70E-08	Included	0.99	0.35
rs8192473	42,299,399	3	C	T	CCK	-0.035 (0.006)	3.58E-09	Included	0.999	0.10
rs11130319	52,755,592	3	A	T	NEK4	0.023 (0.003)	3.72E-11	Included	0.99	0.47
rs4686392	185,524,081	3	A	G	IGF2BP2	-0.032 (0.004)	1.42E-18	Included	0.999	0.33
rs1996023	45,164,637	4	T	G	GNPDA2,GABRG1	-0.032 (0.004)	1.06E-17	Included	0.999	0.28
rs1035491	63,962,177	5	A	G	RGS7BP,FAM159B	-0.024 (0.004)	5.63E-09	Included	0.94	0.27
rs6881648	74,991,849	5	A	C	POC5	-0.024 (0.003)	2.03E-12	Included	0.99	0.43
rs1846974	87,969,927	5	G	A	LINC00461	0.027 (0.003)	1.81E-15	Included	0.996	0.47
rs10062657	95,867,908	5	C	A	PCSK1	-0.038 (0.004)	3.89E-25	Included	0.91	0.40
rs4308481	122,652,106	5	C	T	PRDM6,CEP120	-0.021 (0.004)	9.71E-09	Excluded[c]	-	-
rs4357030	124,316,031	5	C	T	ZNF608,LOC101927421	0.024 (0.004)	3.60E-10	Included	0.88	0.46
rs329120	133,861,756	5	C	T	JADE2	-0.019 (0.004)	3.70E-08	Included	0.999	0.38
rs35261542	20,675,792	6	C	A	CDKAL1	-0.039 (0.004)	3.61E-29	Included	0.99	0.41
rs183975233	32,437,160	6	T	A	HLA-DRA,HLA-DRB5	-0.031 (0.004)	7.51E-16	Included	0.999	0.38
rs6913361	34,179,390	6	A	G	GRM4,HMGA1	-0.038 (0.005)	2.73E-14	Included	0.98	0.13
rs2206271	50,786,008	6	T	A	TFAP2B	0.031 (0.004)	2.77E-18	Included	0.98	0.35
rs148546399	64,705,610	6	G	A	EYS	0.050 (0.008)	1.13E-09	Included	0.75	0.04
rs9397585	153,396,875	6	T	C	RGS17	0.021 (0.004)	5.05E-09	Included	0.99	0.36
rs6947395	69,406,661	7	A	T	AUTS2	0.029 (0.004)	4.81E-12	Included	0.99	0.19
rs143665886	115,368,366	7	T	C	LINC01392,TFEC	0.022 (0.004)	9.46E-09	Included	0.99	0.43
rs77636220	64,552,779	8	G	A	LOC102724612,LINC01289	0.028 (0.004)	2.80E-11	Included	0.99	0.21
rs28857569	76,697,034	8	T	C	HNF4G,LINC01111	0.023 (0.004)	2.00E-09	Included	0.98	0.31
rs4366055	95,507,328	8	A	C	KIAA1429	-0.020 (0.003)	2.12E-09	Included	0.99	0.49
rs7020996	22,129,579	9	C	T	CDKN2B-AS1,DMRTA1	0.032 (0.004)	5.87E-18	Included	0.999	0.44
rs10868215	87,234,111	9	T	C	SLC28A3,NTRK2	-0.021 (0.004)	1.34E-08	Included	0.999	0.32
rs3932549	97,073,588	9	A	C	ZNF169,NUTM2F	0.025 (0.004)	1.97E-09	Included	0.99	0.30
rs5015933	128,137,418	9	T	C	GAPVD1,MAPKAP1	-0.021 (0.003)	6.12E-10	Included	0.99	0.46
rs10795945	12,302,607	10	T	C	CDC123,CAMK1D	0.021 (0.003)	1.10E-09	Included	0.995	0.45
rs7912454	18,584,792	10	A	G	CACNB2	-0.029 (0.005)	3.62E-10	Included	0.98	0.17
rs80117551	69,834,828	10	C	T	HERC4	-0.022 (0.004)	1.57E-08	Included	0.92	0.25
rs1832886	94,477,539	10	G	A	HHEX,EXOC6	0.031 (0.004)	2.59E-12	Included	0.995	0.20
rs12569457	99,096,676	10	C	T	FRAT2,RRP12	0.025 (0.004)	6.67E-09	Included	0.99	0.19
rs2495707	102,425,949	10	A	G	HIF1AN,PAX2	-0.025 (0.004)	1.21E-09	Included	0.90	0.46
rs4409766	104,616,663	10	T	C	C10orf32-ASMT	0.025 (0.004)	5.08E-11	Included	0.999	0.29
rs7903146	114,758,349	10	C	T	TCF7L2	-0.056 (0.008)	9.41E-12	Included	1.000	0.04
rs1907240	122,897,959	10	G	A	MIR5694,FGFR2	-0.024 (0.004)	3.47E-11	Included	0.999	0.31
rs1568079	125,251,751	10	T	A	BUB3,GPR26	-0.025 (0.004)	1.80E-12	Included	0.992	0.36
rs60808706	2,857,233	11	G	A	KCNQ1	0.046 (0.004)	1.24E-38	Included	0.97	0.39
rs16937956	8,404,501	11	A	G	LMO1,STK33	-0.022 (0.003)	5.16E-11	Included	1.000	0.43
rs11030100	27,677,586	11	G	T	BDNF	-0.038 (0.003)	1.19E-28	Included	0.992	0.40
rs11602339	47,761,471	11	C	T	FNBP4	0.023 (0.004)	1.01E-09	Included	0.98	0.32
rs80234489	31,441,179	12	A	C	FAM60A	-0.031 (0.005)	1.05E-11	Included	0.96	0.19
rs3205718	50,261,809	12	C	T	FAIM2	0.023 (0.004)	4.62E-10	Included	0.98	0.28
rs77511173	53,883,537	12	T	C	MAP3K12	-0.044 (0.008)	1.46E-08	Excluded	-	-
rs7305242	112,256,762	12	T	C	ALDH2,MAPKAPK5-AS1	-0.021 (0.004)	2.21E-08	Included	1.000	0.37
rs9568867	54,107,352	13	G	A	LINC01065,LINC00558	0.031 (0.004)	1.03E-14	Included	0.99	0.22
rs75766425	52,511,911	14	G	C	NID2	0.034 (0.005)	1.28E-10	Included	0.97	0.13
rs729050	94,109,502	14	G	T	UNC79	0.021 (0.003)	2.08E-09	Included	0.995	0.41
rs2593235	57,541,201	15	G	A	TCF12	-0.020 (0.003)	1.60E-08	Included	0.99	0.39
rs72749754	62,319,432	15	G	C	VPS13C	-0.026 (0.004)	7.68E-10	Included	0.99	0.23
rs2540034	4,022,694	16	C	T	ADCY9	0.028 (0.004)	2.97E-12	Included	0.87	0.33
rs12597682	20,258,432	16	C	A	GPR139,GP2	-0.030 (0.004)	8.25E-12	Included	0.96	0.19
rs62034325	28,538,640	16	A	G	IL27,NUPR1	0.032 (0.005)	3.81E-10	Included	0.99	0.12
rs11642015	53,802,494	16	C	T	FTO	0.081 (0.004)	2.04E-81	Included	0.99	0.20
rs4788694	73,070,083	16	C	G	ZFHX3	-0.021 (0.004)	2.54E-08	Included	0.91	0.32
rs180950758	29,036,425	17	A	T	SUZ12P1	0.027 (0.005)	2.63E-08	Excluded[c]	-	-
rs4790981	65,921,834	17	A	G	BPTF	0.024 (0.004)	4.65E-10	Included	0.98	0.30
rs1518170	40,708,905	18	T	C	RIT2,SYT4	-0.021 (0.004)	6.80E-09	Included	0.99	0.31
rs6567160	57,829,135	18	T	C	PMAIP1,MC4R	0.052 (0.004)	8.44E-37	Included	1.000	0.20
rs35560038	46,175,046	19	A	T	GIPR	-0.054 (0.004)	2.83E-52	Included	0.97	0.38
rs16978956	18,288,165	20	A	G	ZNF133	0.026 (0.004)	3.46E-09	Included	0.99	0.19
rs2247627	54,145,086	20	G	A	LINC01441,CBLN4	0.019 (0.003)	2.54E-08	Included	0.97	0.45
rs6089584	60,564,086	20	G	C	TAF4	-0.022 (0.004)	3.58E-09	Included	0.98	0.34
rs9983113	40,315,316	21	G	T	LOC400867,LOC101928435	-0.023 (0.004)	2.32E-08	Included	0.95	0.24
rs139913	40,713,861	22	T	A	TNRC6B	-0.027 (0.003)	2.29E-15	Included	0.996	0.47
rs1379871	31,854,782	X	G	C	DMD	0.018 (0.003)	1.05E-08	Excluded	-	-
rs6529684	53,542,107	X	A	G	HSD17B10,HUWE1	0.016 (0.003)	2.78E-08	Excluded	-	-
rs3121672	117,916,370	X	T	C	IL13RA1	0.024 (0.003)	2.90E-17	Excluded	-	-
rs1190736	136,113,464	X	C	A	GPR101	-0.017 (0.003)	1.31E-08	Excluded	-	-
rs5945324	152,894,551	X	G	C	FAM58A,DUSP9	0.022 (0.003)	1.33E-11	Excluded	-	-

Data are derived from Akiyama et al. (Nat Genet 2017; 49: 1458–67). Beta coefficients, standard errors (SE), and *P*-values are the results of a meta-analysis of the same study.

GRS, genetic risk score.

[a]Positions are based on GRCh37/hg19.

[b]Alternative alleles were treated as effective alleles.

[c]Alleles excluded due to lack of availability.
