# Supplementary material for: Effects of gene–lifestyle interactions on obesity based on a multi-locus risk score: A cross-sectional analysis
Source: PLoS One. 2023 Feb 8;18(2):e0279169. doi: 10.1371/journal.pone.0279169 (PMC9907830; doi:10.1371/journal.pone.0279169)
Supplement: S2 Table — (PDF) [file pone.0279169.s004.pdf]

S2 Table. Number of participants recruited at each site.

Recruited site (%)	Total participants (n = 12,918)	Subgroups according to genetic risk score	
		Lower half (n = 6,461)	Upper half (n = 6,457)
Aichi 1	1082 (8.4)	537 (8.3)	545 (8.4)
Aichi 2	1003 (7.8)	494 (7.6)	509 (7.9)
Aichi 3	1888 (14.6)	934 (14.5)	954 (14.8)
Chiba	1009 (7.8)	520 (8.0)	489 (7.6)
Fukuoka 1	1461 (11.3)	719 (11.1)	742 (11.5)
Fukuoka 2	959 (7.4)	489 (7.6)	470 (7.3)
Kagoshima	1094 (8.5)	560 (8.7)	534 (8.3)
Kyoto	1016 (7.9)	521 (8.1)	495 (7.7)
Saga	1803 (14.0)	893 (13.8)	910 (14.1)
Shiga	466 (3.6)	232 (3.6)	234 (3.6)
Shizuoka	508 (3.9)	267 (4.1)	241 (3.7)
Tokushima	629 (4.9)	295 (4.6)	334 (5.2)
