# Supplementary material for: Effects of gene–lifestyle interactions on obesity based on a multi-locus risk score: A cross-sectional analysis
Source: PLoS One. 2023 Feb 8;18(2):e0279169. doi: 10.1371/journal.pone.0279169 (PMC9907830; doi:10.1371/journal.pone.0279169)
Supplement: S3 Table — (PDF) [file pone.0279169.s005.pdf]

S3 Table. Sensitivity analysis excluding participants recruited at Cancer Center.

Parameters				
Random effects	Variance	Standard deviation		
Recruited sites (intercept)	0.34	0.58		
Residual	7.80	2.79		
Fixed effects		95% confidence interval		
	Coefficient estimate	Lower	Upper	p value
Intercept	23.43	23.07	23.79	< 0.001
GRS (high GRS subgroup)	0.62	0.52	0.72	< 0.001
Age	-0.04	-0.12	0.04	0.319
Sex (female)	-1.40	-1.51	-1.28	< 0.001
Protein	0.14	0.06	0.22	0.000
Saturated fatty acids	-0.04	-0.13	0.05	0.413
n-6 polyunsaturated fatty acids	0.17	0.11	0.23	0.000
Carbohydrate	0.10	0.03	0.16	0.002
Soluble dietary fiber	-0.34	-0.41	-0.27	< 0.001
Retinole	0.07	0.01	0.13	0.018
Vitamin B1	0.09	0.02	0.15	0.007
Age * sex	0.56	0.46	0.67	< 0.001
Genetic risk score * saturated fatty acids	-0.12	-0.22	-0.02	0.021

All continuous variables included as fixed effects are standardized. Variables selected by the backward reduction from the following fixed effects are shown: GRS (low and high GRS subgroups coded as 0 and 1), age, sex, BMI measurement method (calculated from examined or self-reported height and weight coded as 0 and 1), protein, saturated fatty acids, monounsaturated fatty acids, n-3 polyunsaturated fatty acids, n-6 polyunsaturated fatty acids, carbohydrate, soluble dietary fiber, insoluble dietary fiber, retinol, vitamin D, vitamin E, vitamin B1, vitamin B2, folate, vitamin C, iron, and calcium, alcohol intake, moderate-to-vigorous physical activity, sitting time, interaction terms between age and sex, and GRS and age, sex, and each lifestyle factor. GRS, genetic risk score; BMI, body mass index.
