# Supplementary material for: Effects of gene–lifestyle interactions on obesity based on a multi-locus risk score: A cross-sectional analysis
Source: PLoS One. 2023 Feb 8;18(2):e0279169. doi: 10.1371/journal.pone.0279169 (PMC9907830; doi:10.1371/journal.pone.0279169)
Supplement: S4 Table — (PDF) [file pone.0279169.s006.pdf]

S4 Table. Characteristics of the subgroups of participants according to BMI and sex.

		Subgroups according to BMI and sex*			
		Normal weight		Obese	
	Total particiants	Male (subgroup 1)	Female (subgroup 2)	Male (subgroup 3)	Female (subgroup 4)
	n = 12,918	n = 3,952	n = 5,221	n = 1,683	n = 1,262
Age (years)	54.7 ± 9.3	55.6 ± 9.3	54.0 ± 9.3	54.7 ± 8.9	56.4 ± 8.6
BMI (kg/m^2)	22.9 ± 3.0	22.4 ± 1.6	21.7 ± 1.7	27.0 ± 1.7	27.1 ± 1.7
Measurement method (self report, %)	2549 ± 19.7	815 ± 20.6	1072 ± 20.5	289 ± 17.2	208 ± 16.5
Daily nutritional intakes					
Energy (kcal)	1685.1 ± 336.2	1889.9 ± 330.7	1518.2 ± 227.9	1881.7 ± 339.6	1524.2 ± 246.1
Protein (g)	52.7 ± 7.0	50.9 ± 7.4	54.2 ± 6.3	50.9 ± 7.2	54.4 ± 6.8
Saturated fatty acid (g)	11.2 ± 2.5	10.4 ± 2.3	11.9 ± 2.5	10.3 ± 2.1	11.6 ± 2.3
Monounsaturated fatty acids (g)	16.0 ± 3.5	15.2 ± 3.5	16.6 ± 3.3	15.5 ± 3.6	17.0 ± 3.6
n3-polyunsaturated fatty acids (g)	2.2 ± 0.5	2.1 ± 0.5	2.3 ± 0.4	2.2 ± 0.6	2.4 ± 0.5
n6-polyunsaturated fatty acids (g)	10.9 ± 2.7	10.2 ± 2.7	11.4 ± 2.6	10.4 ± 2.9	11.7 ± 2.9
Carbohydrate (g)	240.6 ± 23.4	240.0 ± 28.1	240.5 ± 18.3	239.6 ± 29.2	244.3 ± 17.3
Total dietary fiber (g)	10.5 ± 2.8	9.5 ± 2.5	11.4 ± 2.8	9.2 ± 2.3	11.2 ± 2.7
Soluble dietary fiber (g)	1.9 ± 0.6	1.7 ± 0.5	2.1 ± 0.6	1.7 ± 0.5	2.0 ± 0.5
Insoluble dietary fiber (g)	7.6 ± 2.0	6.9 ± 1.7	8.3 ± 2.0	6.7 ± 1.6	8.2 ± 1.9
Retinol (mcg)	923.7 ± 360.6	851.1 ± 337.9	988.9 ± 363.1	833.4 ± 346.0	1002.5 ± 375.7
Vitamin B1 (mg)	7.1 ± 2.9	6.9 ± 3.1	7.3 ± 2.6	6.9 ± 3.2	7.6 ± 2.8
Vitamin B2 (mg)	8.0 ± 1.8	7.5 ± 1.7	8.4 ± 1.8	7.5 ± 1.8	8.6 ± 1.8
Folate (mcg)	0.6 ± 0.1	0.6 ± 0.1	0.7 ± 0.1	0.6 ± 0.1	0.7 ± 0.1
Vitamin C (mg)	1.1 ± 0.2	1.0 ± 0.2	1.1 ± 0.2	1.0 ± 0.2	1.1 ± 0.2
Vitamin D (mcg)	326.0 ± 94.6	300.7 ± 84.3	349.5 ± 97.0	290.8 ± 82.0	351.6 ± 95.9
Vitamin E (mg)	94.3 ± 33.8	83.9 ± 28.5	103.9 ± 34.9	80.6 ± 27.5	104.0 ± 35.0
Calcium (mg)	505.1 ± 139.2	467.9 ± 128.8	542.0 ± 140.6	455.3 ± 116.1	526.0 ± 136.5
Iron (mg)	6.9 ± 1.7	6.5 ± 1.7	7.3 ± 1.7	6.4 ± 1.7	7.3 ± 1.7
Ethanol (g)	13.4 ± 22.5	24.6 ± 27.4	4.1 ± 9.0	26.0 ± 29.1	3.1 ± 8.0
Physical activity (METs-h/day)	13.7 ± 12.5	13.7 ± 12.7	13.4 ± 11.6	13.6 ± 14.0	15.1 ± 13.5
Sitting time (hours/day)	4.9 ± 3.7	5.4 ± 3.9	4.6 ± 3.5	5.2 ± 3.9	4.2 ± 3.5
Generic risk score	-0.07 ± 0.16	-0.1 ± 0.2	-0.1 ± 0.2	-0.0 ± 0.2	-0.0 ± 0.2

Data are shown as means ± SD, unless otherwise specified.

*Normal weight is defined as BMI > 18.5 kg/m^2, and < 25 kg/m^2. Obese is defined as BMI ≥ 25 kg/m^2.

BMI, body mass index; mcgRE, retinol equivalent; METs-h/day, metabolic equivalents-hours per day.
