# Supplementary material for: Effects of gene–lifestyle interactions on obesity based on a multi-locus risk score: A cross-sectional analysis
Source: PLoS One. 2023 Feb 8;18(2):e0279169. doi: 10.1371/journal.pone.0279169 (PMC9907830; doi:10.1371/journal.pone.0279169)
Supplement: S5 Table — (PDF) [file pone.0279169.s007.pdf]

S5 Table. Subgroup analysis by GRS for the candidate approach.

	Subgroups according to GRS															
Parameters	First quartile				Second quartile				Third quartile				Fourth quartile			
Numer of participants	n = 3,227				n = 3,234				n = 3,227				n = 3,230			
Random effects	Variance	Standard error			Variance	Standard error			Variance	Standard error			Variance	Standard error		
Recruited sites (intercept)	0.36	0.60			0.31	0.56			0.31	0.55			0.39	0.63		
Residual	7.18	2.68			7.68	2.77			8.12	2.85			8.08	2.84		
Fixed effects		95% confidence interval				95% confidence interval				95% confidence interval				95% confidence interval		
	Estimate	Lower	Upper	P value	Estimate	Lower	Upper	P value	Estimate	Lower	Upper	P value	Estimate	Lower	Upper	P value
Intercept	23.13	22.75	23.50	< 0.001	23.51	23.16	23.87	< 0.001	23.73	23.38	24.08	< 0.001	24.26	23.87	24.65	< 0.001
Age (years)	0.03	-0.12	0.18	0.691	0.07	-0.08	0.22	0.337	-0.08	-0.24	0.08	0.311	-0.18	-0.33	-0.02	0.029
Sex (female)	-1.28	-1.48	-1.08	< 0.001	-1.37	-1.58	-1.17	< 0.001	-1.31	-1.52	-1.09	< 0.001	-1.44	-1.66	-1.22	< 0.001
Protein	-				-				0.43	0.25	0.61	< 0.001	0.19	0.06	0.32	0.004
Saturated fatty acids	-				-				-0.25	-0.39	-0.12	< 0.001	-			
n-3 poly unsaturated fatty acids	-				-				0.21	0.08	0.35	0.002	-			
n-6 poly unsaturated fatty acids	0.20	0.09	0.30	< 0.001	0.22	0.10	0.33	< 0.001	-				-			
Carbohydrate	-				0.15	0.05	0.25	0.004	-				0.14	0.04	0.25	0.004
Soluble dietary fiber	-0.30	-0.42	-0.18	< 0.001	-0.35	-0.47	-0.23	< 0.001	-0.30	-0.42	-0.17	< 0.001	-0.26	-0.40	-0.12	< 0.001
Retinole	0.18	0.07	0.29	0.001	-				-				-			
Vitamin D	-				-				-0.29	-0.46	-0.12	< 0.001	-			
Vitamin E	-				-				-				0.19	0.06	0.31	0.003
Vitamin B1	-				0.27	0.16	0.37	< 0.001	-				-			
Calcium	-				-				-				-0.21	-0.34	-0.08	0.002
Age * sex	0.49	0.30	0.67	< 0.001	0.53	0.33	0.72	< 0.001	0.71	0.51	0.91	< 0.001	0.59	0.39	0.79	< 0.001

Hyphens indicate variables that were eliminated in the variable selection procedure. GRS, genetic risk sore; BMI, body mass index.
