# Supplementary material for: Effects of gene–lifestyle interactions on obesity based on a multi-locus risk score: A cross-sectional analysis
Source: PLoS One. 2023 Feb 8;18(2):e0279169. doi: 10.1371/journal.pone.0279169 (PMC9907830; doi:10.1371/journal.pone.0279169)
Supplement: S6 Table — (PDF) [file pone.0279169.s008.pdf]

S6 Table. Beta coefficients and *P*-values for the interaction analysis[a] in the candidate approach.

	Beta coefficients			
Candidate variable	GRS	Candidate variable	Interaction	*P*-value for interaction
Protein	2.486	0.044	-0.155	0.32
Saturated fatty acids	2.486	-0.077	-0.411	0.01
n-3 polyunsaturated fatty acids	2.477	0.119	-0.091	0.56
n-6 polyunsaturated fatty acids	2.474	0.099	-0.060	0.70
Carbohydrate	2.496	0.079	0.247	0.11
Retinole	2.492	-0.011	-0.203	0.18
Vitamin D	2.490	0.011	-0.153	0.32
Vitamin E	2.484	0.049	-0.063	0.69
Vitamin B1	2.491	0.088	0.070	0.65
Calcium	2.495	-0.152	-0.461	0.003

Candidate variables were standardized in each model. Beta coefficients for GRS are per one risk allele increase, and those for candidate variables are per one standardized deviation increase in the intake.

[a]Gene–lifestyle interactions were analyzed by the linear-mixed model with BMI as a dependent variable, a recruited site-specific random intercept, and the fixed effects of age, sex, GRS, interaction between age and sex, and interaction between GRS and candidate variable. GRS, genetic risk score.
