# Supplementary material for: Effects of gene–lifestyle interactions on obesity based on a multi-locus risk score: A cross-sectional analysis
Source: PLoS One. 2023 Feb 8;18(2):e0279169. doi: 10.1371/journal.pone.0279169 (PMC9907830; doi:10.1371/journal.pone.0279169)
Supplement: S1 Appendix — (PDF) [file pone.0279169.s009.pdf]

S1 Appendix

Supplementary methods

Statistical analysis

After the gene–lifestyle interaction analysis by the simultaneous modeling approach, we further explored the interaction effects using a candidate approach. A total of 12,918 participants that were analyzed by the simultaneous approach, with 6,461 and 6,457 participants in lower and upper genetic risk score (GRS) halves, respectively, were further divided into four subgroups with 3,227, 3,234, 3,227, and 3,230 participants in the first, second, third, and fourth quartiles, respectively. A linear-mixed model was constructed using the same methods used for the simultaneous approach (see the main manuscript). For the candidate approach, the dependent variable was BMI, with a recruited site-specific random intercept, and the fixed effects were age, sex, BMI measurement method (calculated from examined or self-reported height and weight coded as 0 and 1), 21 lifestyle factors, and interaction terms between age and sex. Variable selection was also performed by the same method used for the simultaneous approach. The highest VIF among all variables in the four models was 2.3, indicating that multicollinearity does not influence the model results. Among the dependent variables selected in each subgroup, candidate variables for the interaction analysis were those that showed inequality in effects on BMI according to the GRS subgroup or variables that were observed not in all subgroups. To analyze the interaction, each candidate variable was assessed separately in respective models with a dependent variable, such as BMI, a recruited site-specific random intercept, and the fixed effect as age, sex, GRS, interaction term between age and sex, and interaction term between GRS and candidate variables.
