# Supplementary material for: Effects of gene–lifestyle interactions on obesity based on a multi-locus risk score: A cross-sectional analysis
Source: PLoS One. 2023 Feb 8;18(2):e0279169. doi: 10.1371/journal.pone.0279169 (PMC9907830; doi:10.1371/journal.pone.0279169)
Supplement: Attachment — Submitted filename: renamed_89f45.docx [file pone.0279169.s010.docx]

**Manuscript: Effects of gene–lifestyle interactions on obesity based on a multi-locus risk score: cross-sectional analysis**

**General and Scientific comments:**

Good job researching on a complex phenomenon of measuring multi-locus risk score for obesity, considering its relevance in the field of personalized genomics. This topic for the manuscript was very well thought out. It is important to look at the combined effect of gene-lifestyle on BMI and its impact on obesity, as it contributes towards realizing the goals of applying an individual’s personalized gene profile to analyze complex phenotypes. The study provides evidence for an interaction effect between genetic risk score on both nutritional intake and physical activi

It is always a good idea to explore through a national multi-institutional cross-sectional data to test for new hypothesis, as this utilizes data that has had effort and time put in to generate it, thereby decreasing the amount of time and work necessary to generate new data. This is also a great step in moving forward to analyze multiple parameters that may have not been tested earlier. Your overall aim is very interesting as it stems from a logical thought process to generate multi-locus risk score considering the polygenic nature of the genetic interactions. However, the hypothesis if there are any or whether it is a hypothesis free research is not clear in the manuscript.

The manuscript is good for consideration for publication, once the suggestions are incorporated.

My overall suggestions would be:

- To be more descriptive in the introduction and discussion e.g., mentioning about nutrigenomics and its’ application in personalized genomic based preventative therapy. A couple of sentences can be included in the abstract, introduction and discussion. This will add value to the goal of the study and allow for thinking of the "big picture" that could be stem from this kind of research.
- Describe the data a little more clearly rather than simply stating what your figures/tables represent, it will enable to better understand the thought process. This will enable you to interpret your data better and summarize your findings coherently.
- The tables can be presented more precisely and effectively. There is too much information which is not really adding to the overall analysis, e.g., of better presentation is 95% confidence interval can be written in one column rather than splitting it into lower and upper, as this is quite standard and understandable. Representing Mean and SD can be like Mean ± SD rather than Mean (SD), as in the same table in other rows ‘n/ any other parameters’ is written within brackets. The table design can be more simplistic. This will allow your readers to understand the article better. Otherwise on an overall, good analysis and interpretation of statistical data. It can be hard interpreting retrospective data!
- Overall work on the presentation of the figures as well. It will be good to work on the vocabulary of language. There seems to be repeat of words e.g., words like however occur in too many places, synonyms of which can be used. Checking for typo and punctuation errors wherever necessary e.g.,the citations are after the full stop in most of the places. I understand that the authors have used a translation service. It will be better to ensure that the message conveyed is not lost in translation and it is the correct intended one. I will highlight some of these points in the detailed review below.

**Specific stepwise comments on the manuscript:**

**Abstract**

- Line 59- To start with importance of lifestyle and obesity research, like introducing about nutrigenomics and then link it to personalized genomics. This will add value.

**Introduction**

- Same comment as above, to include points about nutrigenomics and then link it to personalized genomics in the first paragraph.
- Line 95- Using ‘contradictory’ rather than ‘controversial’, which is quite a strong word in the context of this area of research.
- Line 101- GRS is not within brackets! It will be better to shift it to line 100 when genetic risk score is first mentioned.
- Line 105- managing obesity is better than the word healthcare. It is too generic a word to be used in this context.

**Materials and methods**

- Line 128- DNA was extracted from buffy coat would be more appropriate.
- Line 160- Previously mentioned method, rather than previously method!
- Line 187 to Line 191- This part is unclear. It is mentioned that a sub-group analysis was performed on sex. However, on line 190 and 191, it mentioned as sex was excluded from sub-group analysis. It would be good to verify on this.

**Results**

- Line 207- Correlation is represented as *r,* using the symbol for rho would be appropriate.
- Table 1- As mentioned earlier, better to write Mean and SD can be like Mean ± SD rather than Mean (SD). In each row the value within the bracket indicates a different parameter. Could probably find better ways to simplify it! Just a suggestion!
- Describe more about the details of what the table 2 and 3 is conveying in the appropriate place.
- Table 3- In the ‘age’ row, the values are in fraction. Not sure why the age is being represented in fraction!

**Discussion**

- Line 299- Change from ‘depending among populations’ to “depending on populations”
- Line 302 & 303- Not clear what is being indicated here, is it the suggestion for the entire findings or just the previous sub-group 4 findings. Also, changing ‘GWAS of the Japanese population’ to “GWAS on the Japanese population” is suggested.
- Line 310 & 312- Not clear interpretation, adding more details will add value. Mentioning contradictory is as controversial gives a very different tone.
- Line 329-Better to replace ‘a relative’ with “relatively”
- Line 339- This sentence “younger participants (40–50 years old) in our analysis of subgroup 3” is misleading. Age 40-50 is considered middle age. If it is in comparison, then better to state relatively younger to the other sub-group!
- Line 347-349- The sentence ‘Other than saturated fatty acids, the age of participants differed slightly among subgroups …..’ can be framed better.
- Line 347-349- The sentence ‘mainly recruited at health checkups,…….’ is not clear. Not sure why past health guidance is mentioned.
