# Supplementary material for: Effects of gene–lifestyle interactions on obesity based on a multi-locus risk score: A cross-sectional analysis
Source: PLoS One. 2023 Feb 8;18(2):e0279169. doi: 10.1371/journal.pone.0279169 (PMC9907830; doi:10.1371/journal.pone.0279169)
Supplement: Attachment — Submitted filename: RESPONSE_TO_REVIEWER_COMMENTS.docx [file pone.0279169.s011.docx]

**RESPONSE TO REVIEWER COMMENTS**

**Reviewer comments:**

**Reviewer #1**:

*Methods:*

*Study population: It will be helpful if the authors mention that the population was from Japan.*

*“local population” is obvious for the authors, but not necessarily for a reader outside of Japan. One needs to infer this. They could use: “participants were residents of Japan who participated…..”*

**Reply:**

Thank you very much for pointing the above point. We have amended line 140 as follows:

“In brief, participants were residents of Japan who participated in health checkups held by local governments, volunteers, or patients recruited at their first visit to the cancer hospital, aged 35 to 69 years.”

*How many patients were assessed from the first visit to the cancer hospital. Individuals coming in for cancer visits may or may not be normal. Might these provide confounding for the results. Will it be useful to undertake analysis without these patients to see if there is an impact of these individuals on the results. If there is no impact of these, the authors may include all cases, but it will be useful to report this, as these patients are likely to be different. It is also possible that these patients are already excluded in the outlier removal undertaken in the methods.*

**Reply:**

We agree with the reviewer that participants recruited at the Cancer Center may cause a bias. We added a sensitivity analysis excluding the 1,082 participants recruited at Aichi Cancer Center. For the Reviewer’s information, the number of participants excluded through the outlier removal procedure was 58.

(line 222) We performed a sensitivity analysis excluding the 1,082 participants recruited at the Cancer Center (Aichi 1, S2 Table).

(line 283) A comparable result was obtained from the sensitivity analysis (S3 Table).

(S3 Table)

*Lines 160-164: The physical activity classification using METs is not clear from the explanation provided here. This reviewer tried to read reference # 25, a prior publication of the same cohort, where the levels seem to be different from 5 described here and not straight forward.*

**Reply:**

Thank you for pointing out the lack of explanation. We have added details of the calculation procedure in the relevant section. We did not include standing time as we intended to assess moderate-to-vigorous physical activity which is higher than 3 METs.

(line 182) The intensity of activity was categorized into five levels: walking, 3.0; and heavy physical work or exercise, 4.5 METs in daily life activities and 3.4, 7.0, and 10.0 METs for leisure‐time activities.

(line 184) We did not include standing time (2.0 METs) as we intended to assess moderate-to-vigorous physical activity (> 3 METs) in our analysis.

*Statistical analysis:*

*Clear and detailed enough to understand the methods. This reviewer is curious to know whether subgroup analysis was performed by sex only. This is frequent in a cohort of this nature, and it will be useful to report even if the results were not informative in such an analysis.*

**Reply:**

Thank you for your suggestion. We have added subgroup analysis according to the sex only in SX1 Table.

*Lines 150-151: Is this the GRS range identified in the cohort? What does the theoretical range mean? If this is indeed the range for the cohort, this should belong to the results section.*

**Reply:**

Thank you for question. The “theoretical range of -2.256 to 2.334” is calculated from previous GWAS (reference number 2). We have amended the relevant sentence for clarity.

(line 168) ]. GRS was constructed with a theoretical range of -2.256 (possessing no predisposition allele) to 2.334 (possessing 152 predisposition alleles), where higher scores indicate a higher genetic predisposition to obesity.

*Lines 195-196: It is not clear what do the authors mean by candidate approach? Does it mean “univariate” v/s multivariable for the final model? The S4 table suggests use of a multivariable model with selection of predictors. It appears to be subgroup analysis by GRS quartiles. What is the goal of this analysis?*

**Reply:**

Thank you for pointing this out. As the reviewer points out, the approach is subgroup analysis by GRS quartiles. This was intended to be part of a sensitivity analysis. We have described the methods in detail.

(line 218) The gene–lifestyle interaction was further explored using a different approach: candidate lifestyle factors were explored in the subgroup analysis according to GRS, followed by interaction analysis factor by factor (candidate approach). The detail of the candidate approach is described in detail in the S1Appendix

*Results:*

*In general, if the 95% CI is provided in addition to the coefficient, standard error is not required in the table. 95% CI is more informative, and it is suggested that the authors stick with this, rather than SD.*

**Reply:**

Thank you for pointing this out. We have amended Tables 2 and 3.

*Lines 208-209: This result can be better reported as: “The increase in BMI for every unit increase in the GRS was 2.45 (95% CI xxx-xxx, p <.001).” When one is using weighted GRS, is the unit of increase per risk allele, or per unit increase in GRS?*

**Reply:**

Thank you for kind advice. We have revised the relevant sentence accordingly. Also, the analysis was based on per unit increase in GRS.

*Table 1: While the table is provided as upper and lower half of GRS, the way to make these subgroups is not clear. Are the 2 subgroups divided at 0? This would be clear if they provided the results of the GRS obtained for the cohort, and clarified that it was divided into 2 groups*

**Reply:**

Thank you for pointing this out. We added the details of the categorization of GRS.

(line 171) The calculated GRS was categorized into lower and upper groups according to the score in order to make the number of participants approximately equal in each group.

*It will be useful to provide an overview summary of the results in the tables in 1 or 2 sentences, e.g.* *there was no difference in the age, sex distribution or the captured environmental variables when compared by the two subgroups of the GRS (Table 1).*

**Reply:**

Thank you for kind suggestion. We have added the following sentences.

(line 230) There were no statistical differences in age, sex distribution, BMI, or the captured environmental variables between the two subgroups of the GRS (Table 1).

(line 251) The only interaction term with GRS and lifestyle factor that remained after the variable selection procedure was saturated fatty acids intake (Table 2).

(line 277) Among the lifestyle factors, gene–lifestyle interactions were observed for n-3 polyunsaturated fatty acids, vitamin B1, and sitting time.

*Lines 216- onwards for Gene-lifestyle interaction analysis: This section should also include some better language description of the results connecting them with the methods.*

*e.g. Using the stepwise regression with backward selection, xxx predictors of relevance were selected for the final model.*

**Reply:**

Thank you for your important suggestion. We have amended the relevant sentence as follows.

(line 241) . The final LMM included 15 predictors of relevance selected via stepwise regression with backward selection: GRS, age, sex, 8 nutritional factors, sitting time, and interaction terms for age × sex, GRS × age, and GRS × saturated fatty acids as fixed effects.

*Line 266: From Table 2, the p-value for the interaction, GRS* saturated fats is 0.016, different from the one in text. Can the authors please verify.*

*Do the authors have any plausible explanation for the interaction between GRS and age?*

*In the high GRS group, every year increase in age decreases the BMI by 0.11 (decrease in 1.1/decade)? While this manuscript is focused on the gene-environment effect, this interaction cannot be completely ignored, and needs at least a comment.*

**Reply:**

Thank you for your pointing this out. We apologize for the errors in Table 2. We have corrected the table.

Also, thank you for the comment about the effect of age. Age was z-score standardized, so 1 unit increase corresponds to approximately 10 years, and the effect of 1 unit increase in age including the interaction effect for the participant in the high GRS group would be, for example, 0.63*1 (GRS) + 0.02*1 (age) + -0.10*1 (interaction) = 0.51/decade, and in the low GRS group would be 0.63*0 (GRS) + 0.02*1 (age) + -0.10*0 (interaction) = 0.02/decade.

*Discussion:*

*This is a cross-sectional study. Hence, the results of the LMM models suggest association. Association is not causation, and it is not appropriate to use the results of the regression models to suggest this.*

*It is understandable that the results are contrary to the hypothesis for saturated fatty acids. As the authors suggest, it is possible that the negative association may reflect that the recruited patients may be already involved in health counseling. However, it is not appropriate to suggest that this association means that individuals with higher GRS would not benefit from counseling for intake of saturated fatty acids. The analysis from this cross-sectional study will not be able to address such a question. They need to incorporate the results in the context of prior literature.*

*The authors have selectively addressed the results of the saturated fats interaction with little attention to other results. It is not clear why this was done. There are other lifestyle factors that were statistically significant in the results that need further attention, literature review and better context in discussion.*

For wider applicability of results, it may be useful to compare the results of this study with similar studies in different race/ethnic groups.

**Reply:**

Thank you for the above comment. We totally agree with Reviewer 1. Accordingly, we revised the manuscript so that readers will not misunderstand that we are indicating a causal relationship. Also we have extensively revised the section following the reviewers comment.

(line 329) Results contradicting the results from prior studies [33, 34] might be due to the differences in ethnicity. Another reason might be the nature of the partial regression coefficient obtained from the analysis, while other predictors were held constant. Although this is the same for other predictor variables, individuals who possess a high GRS and higher BMI could focus on factors associated with BMI described in Table 2 other than saturated fatty acids, such as decreasing energy intake (protein, fats, and carbohydrate), increasing soluble dietary fiber intake and decreasing sitting time, because these are consistent with the known evidence [35, 36]. Incorporating a dietary pattern, which is also indicated in the prior literature, in the future study is an idea to solve challenges in interpreting the partial regression coefficient.

**Reviewer #2:**

**General and Scientific comments:**

*Good job researching on a complex phenomenon of measuring multi-locus risk score for obesity, considering its relevance in the field of personalized genomics. This topic for the manuscript was very well thought out. It is important to look at the combined effect of gene-lifestyle on BMI and its impact on obesity, as it contributes towards realizing the goals of applying an individual’s personalized gene profile to analyze complex phenotypes. The study provides evidence for an interaction effect between genetic risk score on both nutritional intake and physical activity.*

*It is always a good idea to explore through a national multi-institutional cross-sectional data to test for new hypothesis, as this utilizes data that has had effort and time put in to generate it, thereby decreasing the amount of time and work necessary to generate new data. This is also a great step in moving forward to analyze multiple parameters that may have not been tested earlier. Your overall aim is very interesting as it stems from a logical thought process to generate multi-locus risk score considering the polygenic nature of the genetic interactions. However, the hypothesis if there are any or whether it is a hypothesis free research is not clear in the manuscript.*

*The manuscript is good for consideration for publication, once the suggestions are incorporated.*

**Reply:**

We appreciate your kind comments. Following your suggestions and those of the other reviewers, we have extensively revised our manuscript.

*My overall suggestions would be:*

- *To be more descriptive in the introduction and discussion e.g., mentioning about nutrigenomics and its’ application in personalized genomic based preventative therapy. A couple of sentences can be included in the abstract, introduction and discussion. This will add value to the goal of the study and allow for thinking of the "big picture" that could be stem from this kind of research.*

**Reply:**

Thank you for your advice. We have added the following sentences.

(line 115) The existence of gene–environment interactions indicates the importance of utilizing gene–lifestyle interactions in primary preventive intervention to select optimal intervention according to genetic risk. For example, personalized nutrition based on nutrigenomic evidence gives specific dietary advice to each individual based on one’s genetic information, rather than a general recommendation about diet [12].

(line 360) Moreover, the results of the subgroup analyses are important for indicating an interaction effect for the groups that are classified with a combination of population group (sex) and phenotypic information (BMI), which includes basic key tiers considered in personalized therapy [12].

- *Describe the data a little more clearly rather than simply stating what your figures/tables represent, it will enable to better understand the thought process. This will enable you to interpret your data better and summarize your findings coherently.*

**Reply:**

Thank you for your advice. We have added the following sentences.

(line 240) There were no statistical differences in age, sex distribution, BMI, or the captured environmental variables between the two subgroups of the GRS (Table 1).

(line 271) There were differences in BMI between the recruited sites with a variance of 0.39 kg/m^2^. The only interaction term with GRS and lifestyle factor that remained after the variable selection procedure was saturated fatty acids intake (Table 2).

(line 303) Among the lifestyle factors, gene–lifestyle interactions were observed for n-3 polyunsaturated fatty acids, vitamin B1, and sitting time. These interactions were prevalent only in subgroups of female participants.

(line 308) Variances of BMI between the recruited sites were largest in this subgroup. No gene‐lifestyle interaction was observed for subgroup 1, while the association between MVPA and BMI was only observed for this subgroup.

- *The tables can be presented more precisely and effectively. There is too much information which is not really adding to the overall analysis, e.g., of better presentation is 95% confidence interval can be written in one column rather than splitting it into lower and upper, as this is quite standard and understandable. Representing Mean and SD can be like Mean ± SD rather than Mean (SD), as in the same table in other rows ‘n/ any other parameters’ is written within brackets. The table design can be more simplistic. This will allow your readers to understand the article better. Otherwise on an overall, good analysis and interpretation of statistical data. It can be hard interpreting retrospective data!*

**Reply:**

Thank you for your advice and comments. We have amended Table 1 following your suggestion.

- *Overall work on the presentation of the figures as well. It will be good to work on the vocabulary of language. There seems to be repeat of words e.g., words like however occur in too many places, synonyms of which can be used. Checking for typo and punctuation errors wherever necessary e.g.,the citations are after the full stop in most of the places. I understand that the authors have used a translation service. It will be better to ensure that the message conveyed is not lost in translation and it is the correct intended one. I will highlight some of these points in the detailed review below.*

**Reply:**

Thank you for your advice. We have extensively revised the manuscript following your comments.

***Specific stepwise comments on the manuscript:***

***Abstract***

- *Line 59- To start with importance of lifestyle and obesity research, like introducing about nutrigenomics and then link it to personalized genomics. This will add value.*

**Reply:**

Thank you for your advice. We have added the following sentence.

(line 77) . Personalized nutrition, which utilizes evidence from nutrigenomics, such as gene–environment interactions, has been attracting attention in recent years.

***Introduction***

- *Same comment as above, to include points about nutrigenomics and then link it to personalized genomics in the first paragraph.*

**Reply:**

Thank you again for raising this point. We have revised this text as mentioned above.

- *Line 95- Using ‘contradictory’ rather than ‘controversial’, which is quite a strong word in the context of this area of research.*

**Reply:**

Thank you for your kind advise. We have revised the word accordingly.

- *Line 101- GRS is not within brackets! It will be better to shift it to line 100 when genetic risk score is first mentioned.*

**Reply:**

Thank you very much for pointing this out. We have corrected this.

- *Line 105- managing obesity is better than the word healthcare. It is too generic a word to be used in this context.*

**Reply:**

Thank you very much for your kind advice. We have revised the relevant point accordingly.

***Materials and methods***

- *Line 128- DNA was extracted from buffy coat would be more appropriate.*
- *Line 160- Previously mentioned method, rather than previously method!*

**Reply:**

Thank you very much for pointing these out. We have revised the relevant sections accordingly.

- *Line 187 to Line 191- This part is unclear. It is mentioned that a sub-group analysis was performed on sex. However, on line 190 and 191, it mentioned as sex was excluded from sub-group analysis. It would be good to verify on this.*

**Reply:**

Thank you for the above comment. We have revised the sentence as follows.

(line 226) As sex was used as stratifying factor, it was dismissed in the subgroup analysis.

***Results***

- *Line 207- Correlation is represented as r, using the symbol for rho would be appropriate.*
- *Table 1- As mentioned earlier, better to write Mean and SD can be like Mean ± SD rather than Mean (SD). In each row the value within the bracket indicates a different parameter. Could probably find better ways to simplify it! Just a suggestion!*
- *Describe more about the details of what the table 2 and 3 is conveying in the appropriate place.*

**Reply:**

Thank you for these comments. We have revised these points in the relevant sections of the manuscript.

- *Table 3- In the ‘age’ row, the values are in fraction. Not sure why the age is being represented in fraction!*

**Reply:**

Thank you for pointing this out. We have ensured that the current Table 3 does not include fraction in the age row.

***Discussion***

- *Line 299- Change from ‘depending among populations’ to “depending on populations”*

**Reply:**

Thank you for your kind advise. We have revised the word accordingly.

- Line 302 & 303- Not clear what is being indicated here, is it the suggestion for the entire findings or just the previous sub-group 4 findings. Also, changing ‘GWAS of the Japanese population’ to “GWAS on the Japanese population” is suggested.

**Reply:**

Thank you for pointing this out. We have revised the sentence as follows.

(line 378) This suggests that the use of SNPs and beta coefficients reported in the GWAS on the Japanese population contributed to observing the gene–environment interaction in the smallest subgroup.

- Line 310 & 312- Not clear interpretation, adding more details will add value. Mentioning contradictory is as controversial gives a very different tone.

**Reply:**

Thank you for pointing this out. We have revised the sentence as follows. We have also changed the word according to your suggestion.

(line 389) Most previous studies have focused on either nutrition or physical activity, making those studies unable to assess marginal effects that consider confounding effects on each other.

- Line 329-Better to replace ‘a relative’ with “relatively”

**Reply:**

Thank you for your suggestion. We have revised the manuscript accordingly.

- Line 339- This sentence “younger participants (40–50 years old) in our analysis of subgroup 3” is misleading. Age 40-50 is considered middle age. If it is in comparison, then better to state relatively younger to the other sub-group!

**Reply:**

Thank you for your critical comment. We have revised the sentence as follows.

(line 426) Indeed, the association between GRS and BMI was stronger for relatively younger participants (40–50 years old) within subgroup 3 (Figure 2).

- Line 347-349- The sentence ‘Other than saturated fatty acids, the age of participants differed slightly among subgroups …..’ can be framed better.

**Reply:**

Thank you for pointing this out. We have revised the section as follows.

(line 437) As mentioned above, saturated fatty acids were considered to be observing the reverse causation. Another example was seen for the association between BMI and physical activity among the subgroups; participants in subgroups 1 and 4 were slightly older (S3 Table), and this might have resulted in observing the association between physical activity and BMI only in these subgroups, i.e., individuals who have difficulty in locomotion tend to have a low BMI owing to loss of lean body mass in subgroup 4.

- Line 347-349- The sentence ‘mainly recruited at health checkups,…….’ is not clear. Not sure why past health guidance is mentioned.

**Reply:**

Thank you for pointing this out. We have revised the section as follows.

(line 442) In addition, because our study focused on the general population, mainly recruited in conjunction with specific health checkups [47], participants with a higher BMI had a higher chance of requiring past health guidance (specific health guidance) [47] regarding diet or physical activity.
